# Rhinovirus induction of fractalkine (CX3CL1) in airway and peripheral blood mononuclear cells in asthma

**DOI:** 10.1371/journal.pone.0183864

**Published:** 2017-08-31

**Authors:** Nadine Upton, David J. Jackson, Alexandra A. Nikonova, Suzie Hingley-Wilson, Musa Khaitov, Ajerico del Rosario, Stephanie Traub, Maria-Belen Trujillo-Torralbo, Max Habibi, Sarah L. Elkin, Onn M. Kon, Michael R. Edwards, Patrick Mallia, Joseph Footitt, Jonathan Macintyre, Luminita A. Stanciu, Sebastian L. Johnston, Annemarie Sykes

**Affiliations:** 1 Airway Disease Infection Section, National Heart & Lung Institute, Imperial College London, London, United Kingdom; 2 MRC & Asthma UK Centre in Allergic Mechanisms of Asthma, London, United Kingdom; 3 Randall Division of Cell and Molecular Biophysics, Kings College London, London, United Kingdom; 4 Imperial College Healthcare NHS Trust, London, United Kingdom; 5 NRC institute of Immunology FMBA, Moscow, Russian Federation; 6 Mechnikov Research Institute for Vaccines and Sera, Moscow, Russian Federation; 7 Respiratory Infection Section, National Heart and Lung Institute, Imperial College London, London, United Kingdom; Katholieke Universiteit Leuven Rega Institute for Medical Research, BELGIUM

## Abstract

Rhinovirus infection is associated with the majority of asthma exacerbations. The role of fractalkine in anti-viral (type 1) and pathogenic (type 2) responses to rhinovirus infection in allergic asthma is unknown. To determine whether (1) fractalkine is produced in airway cells and in peripheral blood leucocytes, (2) rhinovirus infection increases production of fractalkine and (3) levels of fractalkine differ in asthmatic compared to non-asthmatic subjects. Fractalkine protein and mRNA levels were measured in bronchoalveolar lavage (BAL) cells and peripheral blood mononuclear cells (PBMCs) from non-asthmatic controls (n = 15) and mild allergic asthmatic (n = 15) subjects. Protein levels of fractalkine were also measured in macrophages polarised *ex vivo* to give M1 (type 1) and M2 (type 2) macrophages and in BAL fluid obtained from mild (n = 11) and moderate (n = 14) allergic asthmatic and non-asthmatic control (n = 10) subjects pre and post *in vivo* rhinovirus infection. BAL cells produced significantly greater levels of fractalkine than PBMCs. Rhinovirus infection increased production of fractalkine by BAL cells from non-asthmatic controls (*P*<0.01) and in M1-polarised macrophages (*P*<0.05), but not in BAL cells from mild asthmatics or in M2 polarised macrophages. Rhinovirus induced fractalkine in PBMCs from asthmatic (*P*<0.001) and healthy control subjects (*P*<0.05). Trends towards induction of fractalkine in moderate asthmatic subjects during *in vivo* rhinovirus infection failed to reach statistical significance. Fractalkine may be involved in both immunopathological and anti-viral immune responses to rhinovirus infection. Further investigation into how fractalkine is regulated across different cell types and into the effect of stimulation including rhinovirus infection is warranted to better understand the precise role of this unique dual adhesion factor and chemokine in immune cell recruitment.

## Introduction

Rhinoviruses (RVs) are common cold viruses, associated with the majority of asthma exacerbations [1,2]. Th1 and related (type 1) immune responses are vital in rhinovirus clearance and resolution of virus-induced cell damage [3]. Multiple studies have demonstrated that RV-induced anti-viral type 1 responses are deficient in asthma [4–8], however due to conflicting reports that do not observe impaired anti-viral responses, the underlying mechanisms remain unclear. In asthma, RV propagates Th2 (type 2) mediated airway inflammation, which correlates with high viral load and increased symptom severity [9–12].

RVs predominately infect bronchial epithelial cells and alveolar macrophages (AMs) via cellular proteins, such as intercellular adhesion molecule 1 (ICAM-1), low-density lipoprotein receptor (LDLR) or cadherin-related family member 3 (CDHR3) in a strain-dependent manner [13, 14, 15]. AMs are the most abundant leukocyte population in the alveoli [5, 16] and serve as a first line of defence against foreign invaders in the lungs [17]. A current paradigm is that type 1 and type 2 cytokines polarize alveolar macrophages into an M1 (classically activated) and M2 (alternatively activated) phenotype respectively [18]. M1 macrophages are believed to be a major source of Th1 promoting cytokines and chemokines [18] and may be important for orchestrating clearance of RV during infection. As RV infection in asthma has been associated with propagation of type 2 inflammation [9–12], it has been proposed that there is involvement of M2 cells in this response [19].

Fractalkine is the only member of the CX3C family of chemokines and has been associated with several inflammatory diseases including asthma [20, 21]. Unlike other chemokines, fractalkine has a longer peptide chain, which encodes a mucin-like stalk [22]. Full-length fractalkine protein is expressed on cell membranes of structural cells including human bronchial epithelial cells [23] via its mucin- stalk. This membrane-expressed form of fractalkine binds and captures migrating immune cells including monocytes/macrophages, mast cells, T cells, B cells and NK cells [24]. Upon binding cells via CX3CR1 (fractalkine receptor), membrane-expressed fractalkine is cleaved by ADAM10/17 proteases, inducing leukocyte transmigration into tissues [25]. Cleavage of membrane-expressed fractalkine also yields soluble fractalkine, which acts as a chemoattractant to further recruit CX3CR1^+^ cells. Basal production of fractalkine by macrophages, epithelial and smooth muscle cells is necessary for maintaining recruitment and inflammatory activity of resident macrophages in tissues [26].

Fractalkine has been implicated in the development of allergic inflammation [27], implying involvement in allergen-induced type 2-driven allergic inflammation of the airways in asthma. *In vitro*, fractalkine production by human airway smooth muscle cells contributes to the recruitment of mast cells into the bronchial mucosa [28]. In allergic asthmatics, inhalation of allergen resulted in increased membrane-expressed fractalkine expression in bronchial biopsies and increased soluble fractalkine levels in cell-free bronchoalveolar lavage (BAL) fluid [29]. Despite this, the Th2 cytokines IL-4 and IL-13 inhibited IFN-**γ** and TNF-**α** induced fractalkine production by human airway smooth muscle cells *in vitro* [30]. Thus the role and regulation of fractalkine in asthma is unclear and, in rhinovirus-induced asthma exacerbations, unknown. We hypothesised that fractalkine is up regulated during RV infection as part of a type 1/M1 driven anti-viral response and that these RV-induced increases may be deficient in asthma.

## Results

### Levels of fractalkine secreted by M1 and M2 macrophages infected with RV16

**Fig 1:**
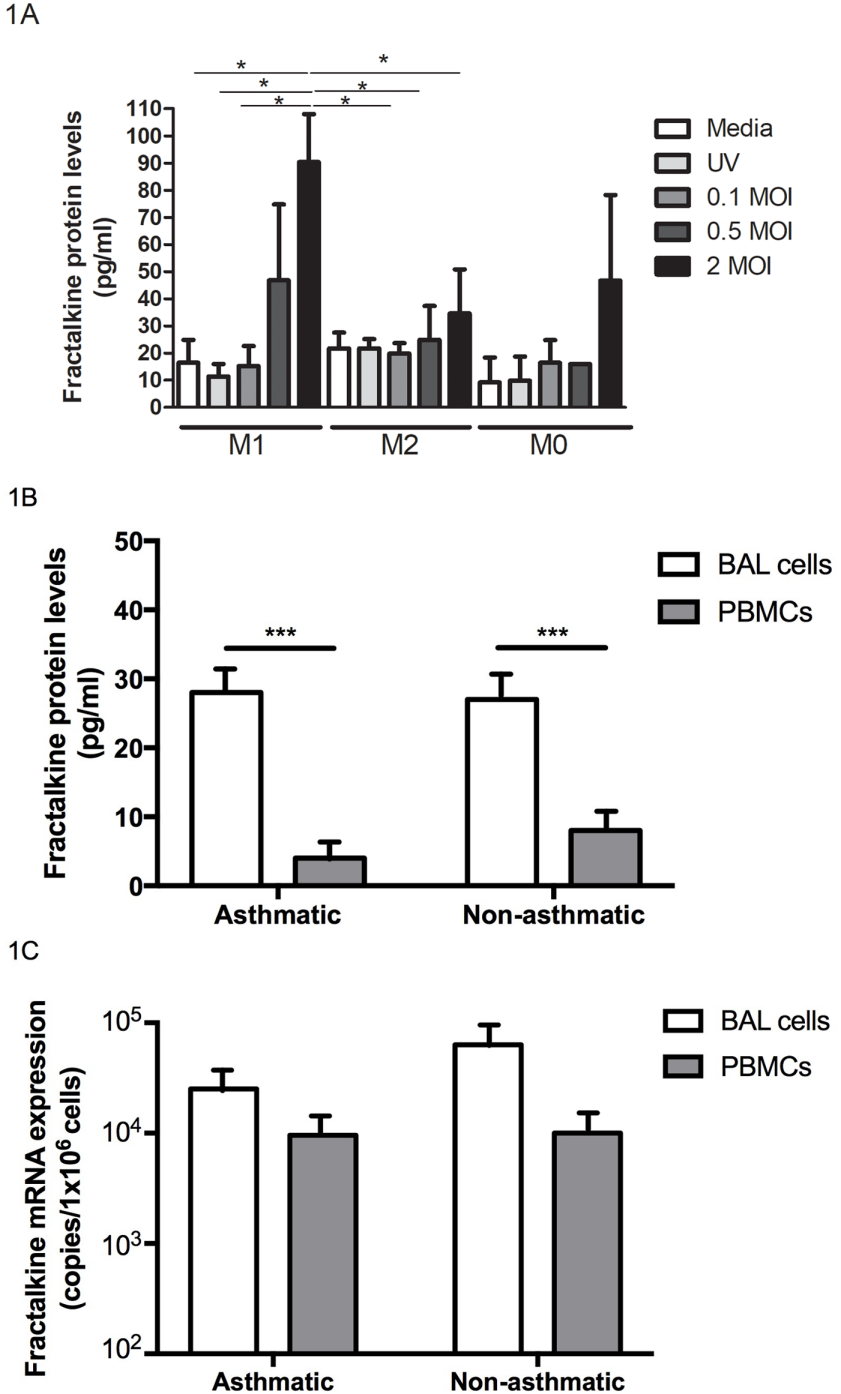
Fractalkine expression *in vitro* by BAL cells, PBMCs and RV16-infected macrophages. A) Soluble fractalkine protein was measured in cell supernatants from human peripheral blood monocyte-derived macrophages (M0) and M1 and M2 polarized macrophages (n = 3) following in vitro RV16 infection (0.1–2 MOI) or control conditions (Media, UK-inactivated RV16). B) Soluble fractalkine protein was measured in PBMC (grey columns) and BAL (white columns) cell supernatants obtained from asthmatic (n = 15) and non-asthmatic (n = 15) subjects following 8h incubation in RPMI medium. C) Gene expression (mRNA levels) was measured in cDNA from PBMC and BAL cells from each subject. All data are expressed as mean ± SEM. Data were analysed by one-way ANOVA with Bonferroni post-test (*P<0.05, ***P<0.001).

To investigate whether secreted fractalkine is a marker of M1 (type 1) or M2 (type 2) macrophages, peripheral blood macrophages (M0) (n = 3) obtained from non-allergic non-asthmatic subjects, were polarised *ex vivo* by incubation with type 1 or type 2 cytokines to give M1 and M2 polarised macrophages respectively as previously described [19]. Fractalkine expression by M1 and M2 macrophages was determined following infection with increasing dosages of rhinovirus RV16 strain. RV16 dose-dependently increased fractalkine production by M1 but not M2 polarized macrophages or M0 macrophages (Fig 1A, *P*<0.05). Fractalkine levels did not significantly differ between non-infected M1 and M2 polarised macrophages.

### Fractalkine levels in BAL cells and PBMCs from asthmatic and non-asthmatic subjects

To compare expression levels of fractalkine produced by non-infected cells, BAL cells and PBMCs from allergic asthmatics (n = 15) and non-asthmatics (n = 15) were incubated for 8h in media alone. BAL cells secreted greater levels of spontaneous soluble fractalkine compared to PBMCs in both non-asthmatic and asthmatic subjects (Fig 1B, *P*<0.001). Fractalkine mRNA was detected at similar levels in both BAL cells and PBMCs (Fig 1C, *P* = NS). There were no differences in protein or mRNA levels between asthmatic and non-asthmatic subjects.

### Effect of *in vitro* RV16 infection on fractalkine levels in BAL cells

**Fig 2:**
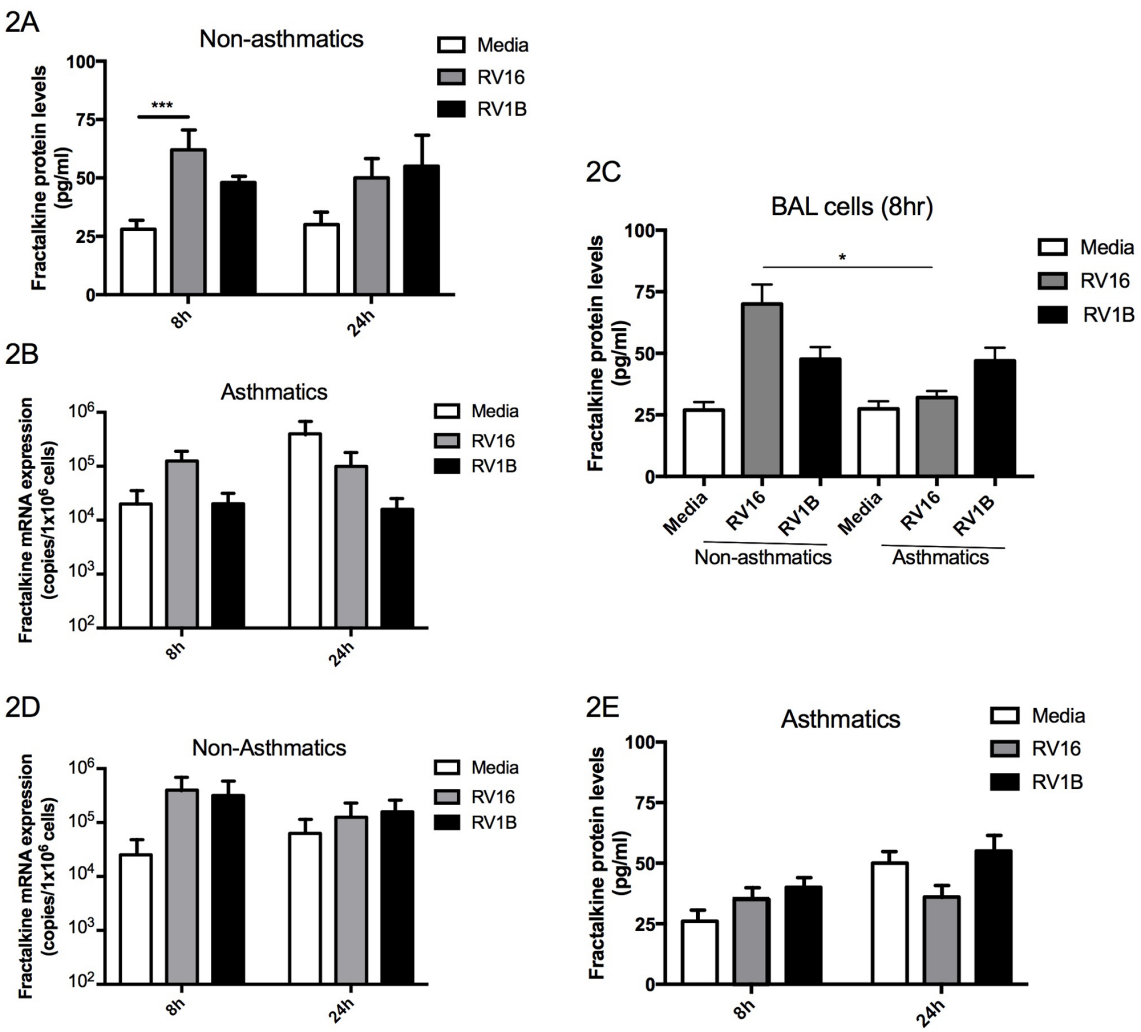
Change in levels of fractalkine in BAL cells following *in vitro* infection with RV16 and RV1B. Soluble fractalkine protein was measured in cell supernatants from BAL cells obtained from (A) non-asthmatic (n = 15) and (B) asthmatic (n = 15) subjects and compared between subject groups at 8hrs post infection (C). Fractalkine mRNA expression was measured in BAL cell lysate cDNA obtained from non-asthmatic (D) and asthmatic (E) subjects. The results are expressed as mean ± SEM. Protein data were analysed by one-way ANOVA with Bonferroni post-test and mRNA by Kruskal Wallis with Dunn’s post test (**P<0.01).

To establish whether *in vitro* RV infection also induces fractalkine expression by BAL cells, protein and mRNA levels of fractalkine were measured at 8h and 24h post RV16 and RV1B infection (major and minor viruses respectively). In non-asthmatics (n = 15), significant increases in fractalkine protein were detected early at 8h following infection with RV16 (Fig 2A, *P*<0.01) but not with RV1B strain. In contrast, in asthmatic subjects, RV infection had no significant effect on fractalkine protein levels (Fig 2B, *P* = NS). Protein levels of fractalkine were significantly greater in non-asthmatic BAL cell supernatants obtained 8hrs post RV16 infection compared to asthmatic BAL cells (P<0.05) (Fig 2C). RV infection had no significant effect on fractalkine mRNA expression in either non-asthmatic (Fig 2D) or asthmatic subjects (Fig 2E). Levels of fractalkine at mRNA level did not differ between asthmatic and non-asthmatic groups.

### Effect of *in vitro* RV16 infection on fractalkine levels in PBMCs

**Fig 3:**
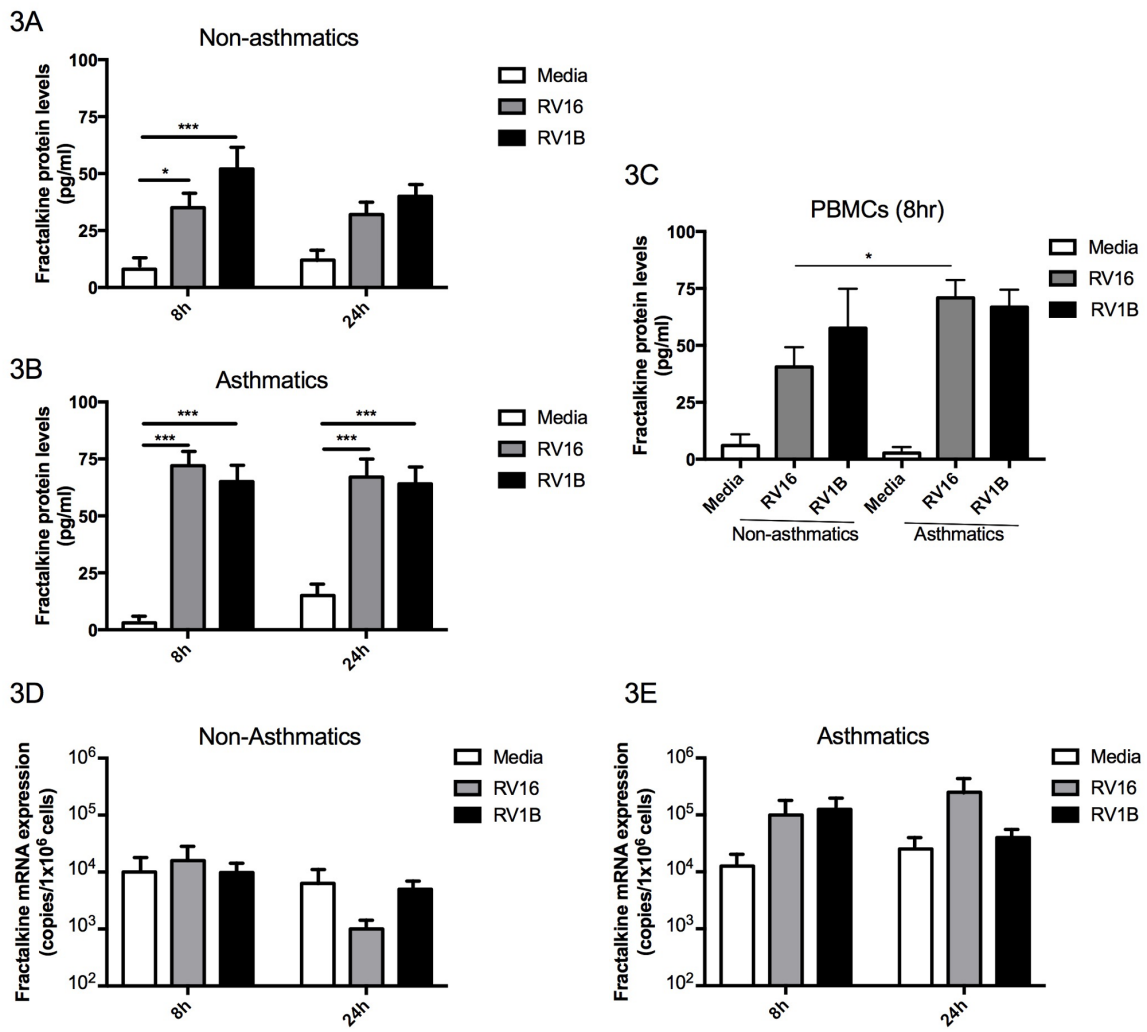
Change in levels of fractalkine in PBMCs following *in vitro* infection with RV16 and RV1B. Soluble fractalkine protein was measured in cell supernatants from PBMCs obtained from (A) non-asthmatic (n = 15) and (B) asthmatic (n = 15) subjects and compared between subject groups at 8hrs post infection (C). Fractalkine mRNA expression was measured in PBMC cell lysate cDNA obtained from (D) non-asthmatic and (E) asthmatic subjects. The results are expressed as mean ± SEM. Protein data were analysed by one-way ANOVA with Bonferroni post-test and mRNA by Kruskal Wallis with Dunn’s post test (*P<0.05, ***P<0.001).

The effect of RV on levels of fractalkine in PBMCs was also assessed at 8h and 24h post RV16 or RV1B infection. Release of soluble fractalkine protein was induced at 8h by RV16 and RV1B in both non-asthmatic (n = 15) (Fig 3A, *P*<0.05 and *P*<0.001 respectively) and in asthmatic (n = 15) groups (Fig 3B, *P*<0.001 for both). Levels of fractalkine were also increased in cell supernatants obtained 24hrs following RV16 or RV1B infection compared to non-infected PBMCs in asthmatics (n = 15) (Fig 3B, *P*<0.001 for both), however this increase was not statistically significant for non-asthmatic subjects (Fig 3A). Levels of fractalkine protein were significantly greater in asthmatic PBMC supernatants collected 8hrs post RV16 infection compared to non-asthmatic PBMCs (P<0.05) (Fig 2C). RV infection had no significant effect on fractalkine mRNA expression in either non-asthmatic (Fig 3D) or asthmatic subjects (Fig 3E). Levels of fractalkine at mRNA level did not differ between asthmatic and non-asthmatic groups.

### Effect of experimental *in vivo* RV16 infection in asthmatic and non-asthmatic subjects on levels of fractalkine in BAL fluid

**Fig 4:**
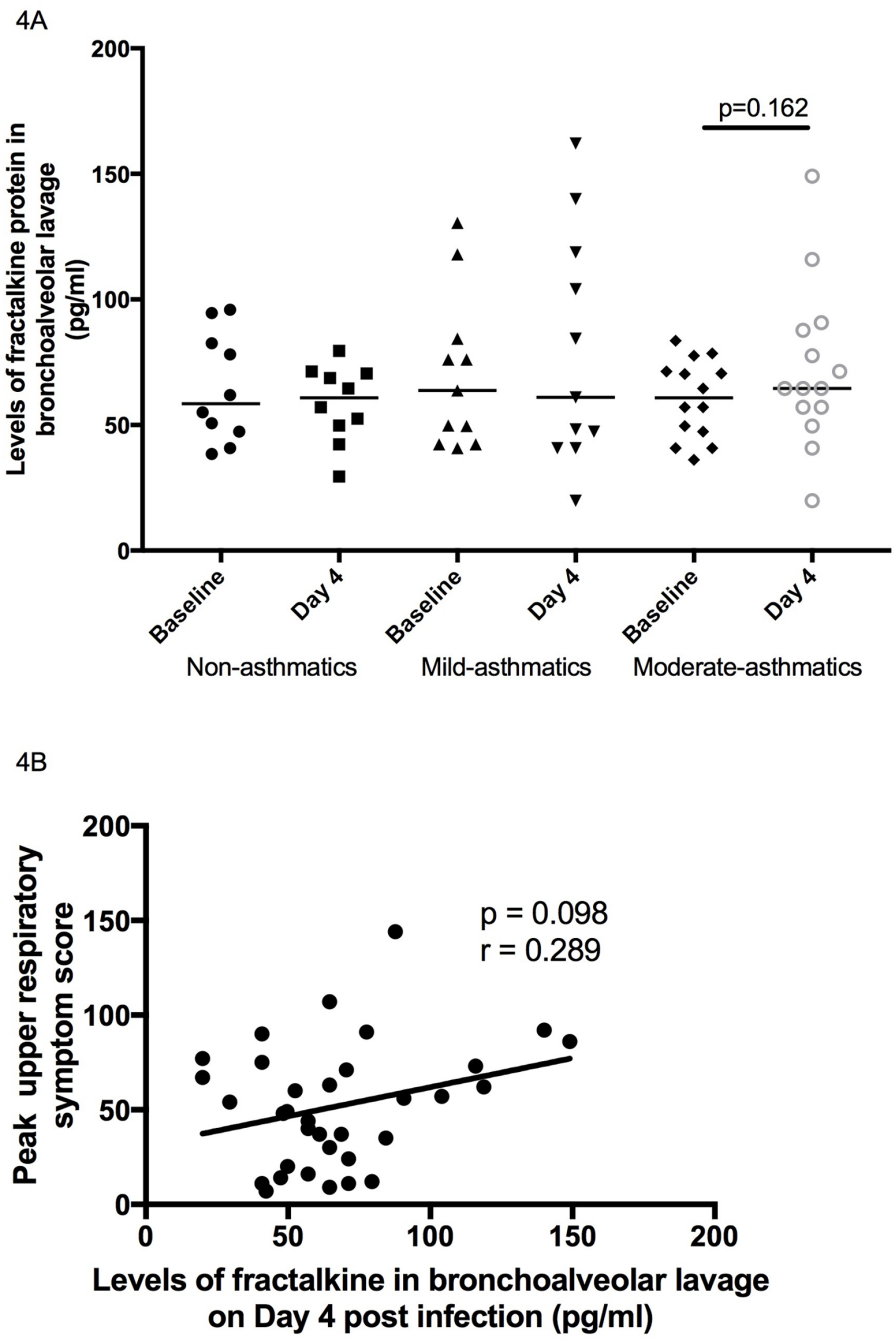
Change in soluble fractalkine in BAL fluid during experimental *in vivo* RV16 infection, related with upper respiratory symptom scores. Soluble fractalkine protein was measured in filtered BAL fluid collected at baseline and day 4 post RV16 infection from non-asthmatic (n = 10), mild-asthmatic (n = 11) and moderate-asthmatic (n = 14) subjects. (A) Data is presented as soluble fractalkine (pg/mL) per subject and horizontal bars for median levels for each group in BAL fluid obtained at baseline and day 4. Data were analysed within groups by Wilcoxon-matched pairs signed rank tests and between groups by Mann Whitney U test, *P<0.05. (B) Levels of fractalkine in BAL fluid on Day 4 were correlated with peak upper respiratory symptom scores for each subject infected using Pearson’s correlation (r = 0.289, *P* = 0.098).

To establish whether RV induces fractalkine protein levels *in vivo*, soluble fractalkine protein was measured in cell-free BAL fluid before (baseline; day -14) and after (day 4) experimental RV16 infection. Levels of fractalkine were similar among all subject groups at baseline (Fig 4). There were no statistically significant differences in levels of fractalkine on day 4-post infection compared to baseline in any subject group (non-asthmatics (n = 10) baseline median 58.50, day 4 60.85, *P*>0.999; mild asthmatics (n = 11) baseline median 63.80, day 4 61.00, *P* = 0.520; moderate asthmatics (n = 14) baseline median 60.85, day 4 64.60, *P* = 0.162). Levels of fractalkine did not statistically differ on day 4-post infection between subject groups (Fig 4). Levels of fractalkine in BAL fluid on day 4 were not significantly related to any clinical or virological outcome after infection, though there was a possible weak relationship with peak upper respiratory symptom scores, however this was not statistically significant (Fig 4B, r = 0.289, *P* = 0.098).

## Discussion

This is the first report for fractalkine responses to RV infection and in asthmatic compared to non-asthmatic subjects. Because previous reports demonstrate fractalkine as a vital dual chemokine and adhesion factor associated with both type 1 and type 2 immune responses [20, 21], we aimed to investigate whether fractalkine was involved in RV-induced immune responses by both mucosal and peripheral blood cells. Our data suggests that fractalkine is produced locally in the bronchial mucosa in response to RV infection as demonstrated by our *in vitro* observation for increased fractalkine production by BAL cells in non-asthmatics following RV infection, however there was no significant induction in asthmatic subjects. This was accompanied by our observation that RV infection induced the production of fractalkine by M1 and not by M2 polarised macrophages. To determine whether this observed impairment was apparent in peripheral blood cells (>70% lymphocytes), we measured levels of fractalkine in PBMCs. We observed that fractalkine is also secreted by PBMCs and that RV increased release of fractalkine in both asthmatic and non-asthmatics and to a greater extent in asthmatics. This suggests fractalkine may also be involved in the activity of circulating immune cells during RV infection, regardless of whether there is induction of a robust anti-viral (type 1) or immunopathological (type 2) immune responses as reported previously for asthmatic and non-asthmatic subjects respectively [4–8]. Our data suggest possible involvement of fractalkine in response to RV infection by multiple cell types and that this dual chemokine/adhesion factor’s specific role may differ in asthmatics compared to non-asthmatics. However, our findings also highlight the complexity of fractalkine regulation *in vivo* and that this may vary across different cell types.

A vital role for alveolar macrophages in early viral clearance has been described previously [19] and BAL cells used for this study produced anti-viral mediators in response to RV infection as previously described [5]. We demonstrate that fractalkine production is increased in response to RV infection by BAL cells from non-asthmatics (Fig 3A), indicating a possible anti-viral role for fractalkine in healthy subjects. Multiple reports describe that macrophages are polarised *in vivo* toward M1/type 1 phenotype following exposure to invading pathogens such as rhinovirus [18, 19] and in this study RV infection of M1 macrophages significantly increased fractalkine production (Fig 1A, *P*<0.05). In line with previous reports for a dominant type 2 immunopathology [9–12] and resulting impaired type 1 anti-viral responses in asthmatics during RV infection [4–8], we report impaired RV-induced increases in fractalkine release from asthmatic BAL cells (Fig 2B). This is accompanied by defective anti-viral interferon production from asthmatic BAL cells as previously described [5]. Mediators associated with anti-viral immune responses to rhinovirus may perhaps be extended to include fractalkine/CX3CL1, a unique dual soluble chemokine and adhesion factor [21], contributing further to our understanding of defective rhinovirus-induced anti-viral responses in asthma.

Here we also demonstrate that RV infection with both major (RV16) and minor (RV1B) strains in PBMCs induced release of fractalkine (Fig 3A and 3B) in both asthmatic and non-asthmatic subjects. It has previously been identified that fractalkine potently induces chemotaxis of CX3CR1^+^ monocytes, natural killer cells and CD4^+^ T cells [23] and, in mouse models of asthma, we have learned that RV infection propagates infiltration of lymphocytes and natural killer cells into the lungs, however the precise chemotaxis axis involved has not yet been determined [31, 32]. As fractalkine induces lymphocyte chemotaxis, [23, 27] and RV infection induced high fractalkine production by PBMCs from asthmatics in our study, it is possible that fractalkine plays a pathogenic role by aiding chemotaxis of circulating CX3CR1^+^ cells involved in type 2 immunopathological responses into the bronchial mucosa during RV infection in asthma. As fractalkine release was also induced in non-asthmatic PBMCs, we could also hypothesise that instead, the role here of fractalkine is to aid chemotaxis of cells involved in type 1 anti-viral responses in non-asthmatics. Unfortunately, as we had a limited availability in this study of BAL cells and PBMCs from subjects, cellular phenotyping by flow cytometry analysis was not performed. Future experiments could phenotype CX3CR1^+^ PBMCs during RV infection to identify cells primed to undergo fractalkine-induced cell migration and assess whether the phenotype of these CX3CR1^+^ cells differ in non-asthmatics versus asthmatics. Our study highlights that fractalkine should be included in future RV and asthma studies to further our understanding for the specific role and impact of this dual chemokine and adhesion factor in recruiting circulating immune cells to sites of infection and to consider the potential effect of the fractalkine-chemotaxis axis on RV-induced symptom severity in asthma.

Following our interesting observations for RV16-induced release of fractalkine by BAL cells and PBMCs were generated from *in vitro* RV infection experiments, we also measured levels of fractalkine in BAL fluid from a separate study [11] to investigate whether *in vivo* RV16 infection increases levels of fractalkine locally in the bronchial mucosa, an important site for RV infection in asthma. We measured soluble fractalkine protein in cell-free BAL fluid from non-asthmatics and mild-moderate asthmatics pre and post *in vivo* RV16 infection. Analysis of inflammatory mediators in BAL fluid before and after RV infection is a tool frequently used to assess environmental changes in the airways during infection. We now report that there were no differences between groups in baseline production of fractalkine, and on day 4 following *in vivo* RV16 infection, levels of fractalkine protein in BAL fluid did not increase from baseline in any group of subjects, though there may have been a very small increase in subjects with moderate asthma, although this increase was not statistically significant (Fig 4A, *P* = 0.162). We next investigated links between fractalkine levels on day 4 and clinical and virological markers of disease severity, as the patients with moderate asthma used in this study experienced greater symptom severity as previously reported [11]. Levels of fractalkine in BAL fluid on day 4 did not relate to any clinical or virological markers of disease severity, though they may appear to relate to peak upper respiratory symptom scores, however this correlation did not reach statistical significance (Fig 4B, *P* = 0.098). It is possible that BAL fluid fractalkine is derived from shedding of membrane-bound fractalkine from bronchial epithelial cells, which may be increased following RV infection, as demonstrated previously *in vitro* [33]. Whether the role of bronchial epithelial-cell derived fractalkine is for recruitment of immune cells in the bronchial mucosa was not determined in this study, however our data does suggest that fractalkine may be important within the bronchial mucosa during a rhinovirus infection *in vivo* in patients with moderate asthma.

Our study has highlighted that fractalkine may play a role during RV infection in a cell- specific manner and that its precise role may differ between non-asthmatic and asthmatic subjects. Our conflicting results indicate that fractalkine levels and activity should be considered in future RV infection studies to better understand the precise kinetics, source and role of this unique dual soluble chemokine and adhesion factor expressed by multiple leukocyte and structural cells relevant in asthma to understand it’s likely role in recruitment of leukocytes to sites of inflammation and regulation of localised anti-viral immune responses in the bronchial mucosa [19–30]. Fractalkine has been considered in other diseases including cardiovascular [34] and neurodegenerative diseases [35] however more attention is needed to define its precise role in respiratory diseases including asthma, in particular during RV infection that’s proven to be a major cause of asthma exacerbations.

## Materials and methods

### Collection and processing of clinical samples

Bronchoscopy and blood collection was performed in 15 non-asthmatics and 15 mild allergic-asthmatics at St Mary’s Hospital, London. Peripheral blood mononuclear cells (PBMCs) and BAL cells (>95% alveolar macrophages) were obtained as previously described [5]. In a separate *in vitro* study, macrophages were obtained from PBMCs from 3 non-allergic-non-asthmatic healthy donors and polarised *in vitro* into M1 or M2 populations. In another clinical study, BAL fluid was collected by bronchoscopy from subjects recruited for a previous experimental *in vivo* RV16 infection study (in which 10 non-allergic-non-asthmatics, 11 mild and 14 moderate allergic-asthmatics were successfully infected with RV16 as described previously) [11]. Bronchoscopy was performed at baseline (~day -14) and day 4-post RV16 infection as previously described [11]. St. Mary’s Hospital Ethics Committee approved all studies. All subjects from both clinical studies gave written informed consent. All human tissues were handled and stored in accordance with Human Tissue Act.

### Viral stocks

RV16 and RV1B serotypes were cultured in Ohio HeLa cells as previously described [5]. Viral stocks were used at a multiplicity of infection (MOI) of 1 for *in vitro* infection experiments.

### Isolation of human monocytes, differentiation into monocyte derived macrophages (MDM) and polarization into M1 and M2 macrophages

PBMCs from 3 non-allergic-non-asthmatic healthy donors, were washed, re-suspended in macrophage serum free media (MSFM, Invitrogen, UK) and seeded into either 6-wells plates (NUNC, Thermo fisher scientific, UK) at 3x10^6^ cells/well or Primaria^™^ tissue culture dishes (Falcon^®^) at 30x10^6^ cells/dish. Non-adherent cells were removed after 2h of incubation at 37°C in a humidified atmosphere containing 5% CO_2_. An equal volume of fresh MSFM containing 10 ng/mL of human Granulocyte-Macrophage Colony Stimulating Factor (GM-CSF, Invitrogen, UK) and penicillin/streptomycin mixture (Invitrogen, UK) were then added to the cells. The cells were differentiated for 7 days. Media was replaced every 3 days. The mature MDM were then stimulated overnight with either type 1 cytokines: 2 ng/mL of TNF-α plus 20 ng/mL of IFN-γ (both from R&D Systems, UK) or with the type 2 cytokine: 20 ng/mL of IL-4 (Invitrogen, UK) to obtain M1 or M2 polarized macrophages, respectively. Un-polarized control, MDM (M0) was maintained in culture overnight in MSFM alone.

### Rhinovirus infection

PBMCs and BAL cells (2x10^6^) from non-allergic-non-asthmatic and allergic asthmatic subjects were isolated and infected *ex vivo* with RV16 or RV1B at a MOI of 1 as previously described [5]. Cell supernatants and lysates were collected at 8 or 24h. Polarized or unpolarized MDMs were treated with live or UV-inactivated RV16 or RV1B [MOI of 0.1, 0.5, 1, 2] for 1h at room temperature. Cells were washed and any non-adherent virus was removed and re-suspended in the fresh media. Cell supernatants and RNA lysates were harvested at the times indicated in the figures and stored at -80°C (the 0h time point means 1h after incubation with RV followed by intensive washing of plates from non-adherent virus and adding of the fresh media). RPMI medium or filtered/UV-treated RV were included as negative controls for all *ex vivo* experiments.

RV16 experimental infections were induced in RV16 seronegative mild and moderate allergic asthmatic and non-allergic-non-asthmatic age-matched subjects as previously described [11]. Briefly, BAL fluid used for this study was obtained by bronchoscopy at baseline (~day -14) and post infection (day 4) and cells removed by filtration.

### RNA isolation, qPCR and Luminex

Total RNA was extracted from BAL cell and PBMC lysates using the RNeasy method (RNeasy Mini Kit; Qiagen, UK) following the manufacturer’s instructions, including DNase digestion (Dnase (Rnase-free Dnase); Qiagen, UK). cDNA was synthesized using Omniscript RT and components as directed by the manufacturer (Qiagen, UK). Conditions for Taqman qPCR reactions used are previously described [5]. For primers and probe sequences, see S1 Table. in supporting information. Soluble fractalkine protein in cell supernatants and BAL fluid was labeled using Luminex milliplex Map kits (Millipore, UK) according to the manufacturer’s instructions and quantified using the Luminex 100 analyzer (Millipore, UK).

### Statistical analysis

Data normality was assessed using the D’Agostino and Pearson omnibus normality test and normally distributed data was presented as mean ± SEM. For time course experiments data was analyzed within groups using one-way ANOVA followed by Bonferroni’s multiple comparison post-test or Kruskal Wallis with Dunn’s post-test. For comparisons between groups, unpaired t-tests were used. Data were accepted as significantly different when *P*<0.05. For data presented as medians, Mann Whitney U test was used to compare between groups, and Wilcoxon matched-pairs signed rank test between time-points within groups. For data correlations, Pearson’s linear regression was performed where *P*<0.05 was considered at significant.

## Supporting information

S1 Table qPCR primer and probe sequences. (DOCX)
