# Supplementary material for: Rhinovirus induction of fractalkine (CX3CL1) in airway and peripheral blood mononuclear cells in asthma
Source: PLoS One. 2017 Aug 31;12(8):e0183864. doi: 10.1371/journal.pone.0183864 (PMC5578648; doi:10.1371/journal.pone.0183864)
Supplement: S1 Table — (DOCX) [file pone.0183864.s001.docx]

**Supporting information**

**S1 Table. qPCR primer and probe sequences**

| **Reagent** | **Sequence** |
| --- | --- |
| Fractalkine Forward (300nM) | 5’ GATACCTGTAGCTTTGCTCATCCA 3’ |
| Fractalkine Reverse (900nM) | 5’ CCAAGATGATTGCGCGTTT 3’ |
| Fractalkine Probe | 5’ TATCAACAGAACCAGGCATCATGCGG 3’ |
| 18S Forward (300nM) | 5’ CGCCGCTAGAGGTGAAATTCT 3’ |
| 18S Reverse (300nM) | 5’ CATTCTTGGCAAATGTCG 3’ |
| 18S Probe | 5’ ACCGGCGCAAGACGGACCAGA 3’ |
